# Left ventricular clefts – incidental finding or pathologic sign of Wilson’s disease?

**DOI:** 10.1186/s13023-019-1238-7

**Published:** 2019-11-07

**Authors:** Kun Zhang, Ulrike Reuner, Marie Weidauer, Uwe Speiser, Karim Ibrahim, Marian Christoph, Frank R. Heinzel, Burkert Pieske, Felix M. Heidrich, Silvio Quick

**Affiliations:** 1 Department of Internal Medicine and Cardiology, Charité – Universitätsmedizin Berlin, Campus Virchow-Klinikum, Augustenburger Platz 1, 13353 Berlin, Germany; 2 Berlin Institute of Health (BIH), Berlin, Germany; 3 Department of Neurology, Technische Universität Dresden, University Hospital, Dresden, Germany; 4 Technische Universität Dresden, Heart Center, University Hospital, Clinic of Internal Medicine and Cardiology, Dresden, Germany; 5 Department of Cardiology, Technische Universität Dresden, Klinikum Chemnitz, Chemnitz, Germany; 6 DZHK (German Centre for Cardiovascular Research), partner site Berlin, Berlin, Germany

**Keywords:** Wilson’s disease, Cardiac magnetic resonance imaging, Left ventricular cleft

## Abstract

**Background:**

Wilson’s disease is an inherited autosomal recessive multi-systemic disorder characterized by reduced excretion and consequently excessive accumulation of copper in different organs, such as the heart.

**Results:**

In a prospective controlled trial, which is the largest to date, we evaluated 61 patients with Wilson’s disease, age- and sex-matched to 61 healthy patients, for cardiac manifestation using cardiac magnetic resonance imaging. Patients were under stable disease and had no signs of heart failure at the time of examination.

We detected a left ventricular cleft, an invagination penetrating more than 50% wall thickness of the adjoining compact myocardium in diastole, in 20% of the patients (12 out of 61) compared to 5% among control patients (3 out of 61, *p* = 0.013). No correlation between the incidence of cleft and a certain genotype of Wilson’s disease was found. All described cases were incidental findings and none of the patients showed other signs of cardiac involvement.

**Conclusions:**

To conclude, the results of this study suggests that the increased occurrence of left ventricular clefts is due to Wilson’s disease. Large studies with a long observation period are needed for further evaluation.

Wilson’s disease is an inherited autosomal recessive multi-systemic disorder characterized by reduced excretion and consequently excessive accumulation of copper in different organs. Cardiac involvement has been considered to be benign in the past. However, cases of cardiac sudden death and an increased incidence of arrhythmias and heart failure have been reported. Just recently, we have demonstrated that even asymptomatic patients exhibit structural disease and autonomic dysfunction of the heart [1, 2].

In a prospective controlled trial, which is the largest to date, we evaluated 61 patients with Wilson’s disease for cardiac manifestation. Wilson’s disease was diagnosed beforehand by hepatic copper content assessment in liver biopsy specimens and laboratory findings (ceruloplasmin level in serum below the limit of normal and 24 h high urinary copper excretion > 100 μg/d). Patients were under stable disease and had no signs of heart failure at the time of examination. All patients underwent cardiac magnetic resonance imaging (cMRI) and were age- and sex-matched to 61 healthy patients.

Intriguingly, we detected a left ventricular cleft, an invagination penetrating more than 50% wall thickness of the adjoining compact myocardium in diastole, in 20% of the patients (12 out of 61) compared to 5% among control patients (3 out of 61, *p* = 0.013). Left ventricular clefts became best visible in the late gadolinium enhancement images and cine sequences in cMRI (Fig. 1, Additional file 1: Video S1). They were located in the septal segments (3 out of 12), the inferior (4 out of 12), the lateral (3 out of 12) and the anterior wall (2 out of 12). Additionally, real-time 3D contrast echocardiography was performed, in which an interventricular shunt could be excluded. No correlation between the incidence of cleft and a certain genotype of Wilson’s disease was found (Table 1).

**Fig. 1:**
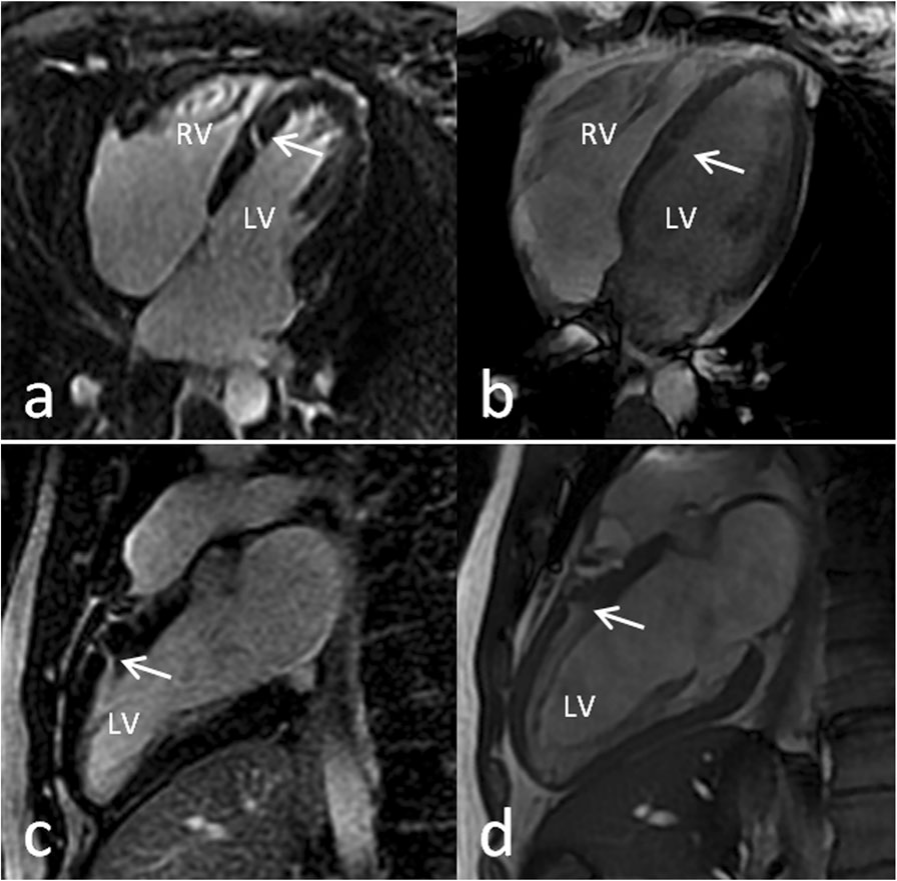
Representative cardiac magnetic resonance images revealing left ventricular clefts. Late gadolinium enhancement images (a and c) and cine sequences (b and d) of two patients with Wilson’s disease. The arrows show a deep left ventricular cleft in the interventricular septum (patient 1, upper row) and in the anterior wall (patient 2, lower row). LV - left ventricle, RV - right ventricle

**Table 1:** Chi-quadrat analysis of Wilson’s disease mutations and left ventricular clefts

Mutation	n_m_	n_c_	*p*-value
H1069Q	24	6	0.39
A1135Q	7	2	0.53
Q1351Stop	5	1	0.98
M769 V	1	1	–
R969Q	2	2	–
P1352S	2	–	–
M769H	5	–	–
R1041W	2	–	–
S105Stop	2	–	–
D765N	2	–	–
H643I	1	–	–
Q111Stop	1	–	–
V845S	1	–	–
G1266R	1	–	–
P1273L	1	–	–
G1061E	1	–	–
T1434 M	1	–	–
L1325R	1	–	–
V1282C	1	–	–

*Nm* number of patients with an underlying mutation for Wilson’s disease, *nc* number of patients with left ventricular clefts


**Additional file 1:** **Video S1.** Video showing interventricular cleft.


Myocardial clefts are congenital abnormalities related to myocardial fiber or fascicle disarray. Congenital myocardial clefts or fissures are commonly seen in the basal inferior wall of the left ventricle and the mid to apical segments of the interventricular septum. Petryka et al. studied 686 patients and found these clefts (also called crypts) to be most prevalent in patients with hypertrophic cardiomyopathy (15.6%), myocarditis (15.3%), and arterial hypertension (13.6%). Clefts were also evident, albeit significantly fewer, in 6.7% of the healthy patients [3]. In other studies, the incidence of clefts in hypertrophic cardiomyopathy was even higher with 61 and 81%, respectively [4, 5]. They were proposed to be a prephenotypic marker of hypertrophic cardiomyopathy, that means representing early signs of myocardial alterations in patients that carry a gene defect who have not displayed the phenotype yet. The high variance in prevalence among different studies may be due to the variability of cMRI procedures and the interpretation thereof.

The results of our study suggest that clefts are due to the pathology of Wilson’s disease. All described cases were incidental findings and none of the patients showed other signs of cardiac involvement. The etiology is a matter of speculation. One hypothesis regarding the pathophysiological mechanism of cleft formation was described by Moon and McKenna who speculate that they may represent a sign of abnormal fetal cardiomorphogenesis [5]. However, it remains unknown how pathological accumulations of copper could support this theory and cause them.

Left ventricular clefts have not been associated to Wilson’s disease before. Previous imaging studies on cardiac manifestation are mainly echocardiography based and have not reported on clefts so far. Likewise, in none of our 12 patients, echocardiography was able to detect any evidence thereof. Echocardiographic visualization can be challenging, particularly if cleft location does not coincide with standard acquisition planes. Therefore, cMRI seems to be more sensitive in detecting structural abnormalities than routine echocardiography.

To conclude, the results of this study suggests the occurrence of left ventricular clefts in cMRI as an early sign of Wilson’s disease in the heart. Large studies with a long observation period are needed for validation. We propose that patients with Wilson’s disease receive cMRI as a standard tool to evaluate cardiac manifestation, specifically with a careful search for left ventricular clefts.

## Data Availability

The datasets used and/or analyzed during the current study are available from the corresponding author on reasonable request.

## Abbreviation

cMRI: Cardiac magnetic resonance imaging
